# Tissue oxygen monitoring leads to lower rates of blood transfusions

**DOI:** 10.1186/cc9845

**Published:** 2011-03-11

**Authors:** A Loukas, C Matadial, J Yapor, R Martinez-Ruiz

**Affiliations:** 1 University of Miami, FL, USA

## Introduction

Evidence exists that blood transfusions may be more harmful for patients than once suspected [1]. Optimal goals for transfusion therapy remain elusive. Lower rates of blood transfusion seem to lead to better patient outcomes. Tissue oxygenation monitoring may offer a novel insight into blood transfusion requirements as it represents an indication of oxygen content further down the oxygen cascade than the blood oxygen content defined by pulse oximetry and hematocrit. Our hypothesis is that the use of this monitor may define a safer, lower threshold for blood transfusion that may lead to decreased transfusion rates.

## Methods

We performed chart reviews of 100 patients who underwent cardiac surgery (coronary artery bypass graft surgery and valvular surgery) with heart-lung bypass. The first 50 surgeries were performed with standard hemodynamic monitors and intraoperative transesophageal echocardiography. Indications for transfusion included ongoing bleeding, hematocrit less than 20% with a heart rate over 95 bpm or blood pressure less than 90 mmHg systolic. The subsequent 50 cases consisted of a similar patient population, surgical indications and medical group; however, the use of the Hutchinson InSpectra tissue oxygen monitor intraoperatively and postoperatively was employed. Our review sought to identify whether the transfusion threshold criteria were modified due to the availability of this additional monitoring information.

## Results

A lower hematocrit value was found to be tolerated as long as tissue oxygen values were within an acceptable range; that is, above 70% or less than a 20% drop from baseline. There was a statistical difference between transfusion rates, in the first group was 30% and in the second group was 18%. This represents a relative decrease of nearly 50% in blood transfusions. Outcomes in both groups were identical. The mortality rate was nil. There was no significant difference between outcomes or length of stay.

## Conclusions

Although optimal goals for blood transfusion remain elusive, it does appear that even slight overtransfusion may be detrimental [1]. The tissue oxygen monitor appears to define a new, lower safe threshold for transfusion. An outcome benefit will probably be observed in future studies. Long-term outcome benefits from the routine implementation of this device have already been suggested in the trauma [2] and intensive care [3] settings.

## References

[B1] Bennett-Guerrero JAMA 2010 304 1568 1575 10.1001/jama.2010.1406 20940382

[B2] Cohn J Trauma 2007 62 44 53 10.1097/TA.0b013e31802eb817 17215732

[B3] Ikossi J Trauma 2007 61 780 790 10.1097/01.ta.0000239500.71419.58 17033541

